# Relationship between palatal bone thickness and maxillary transverse deficiency in skeletal class II adults: A cone-beam computed tomography analysis

**DOI:** 10.4317/jced.63379

**Published:** 2025-11-30

**Authors:** Karina Lisbeth Sánchez-Morillo, Luis Ernesto Arriola-Guillén, Marjory Elizabeth Vaca-Zapata, Yalil Augusto Rodríguez-Cárdenas

**Affiliations:** 1 DDS. Orthodontic student, School of Dentistry, Hemisferios University, Quito, Ecuador; 2 DDS, MSc, PhD. Associate Professor of the Division of Orthodontics and Division of Oral and Maxillofacial Radiology, School of Dentistry, Universidad Scientific del Sur, Lima, Perú; 3 DDS, MSc, Associate Professor of the Division of Orthodontics, School of Dentistry, Hemisferios University, Quito, Ecuador; 4 DDS, MSc, PhD. Assistant Professor of the Division of Oral and Maxillofacial Radiology, Faculty of Dentistry, Universidad Nacional de Colombia, Bogotá D.C, Colombia

## Abstract

**Background:**

Maxillary Transverse Deficiency (MTD) is a condition that affects the balance and functionality of the stomatognathic system. The aim was to evaluate maxillary bone thickness at the level of the midpalatal suture (MPS) using Cone Beam Computed Tomography (CBCT) in skeletal Class II individuals with maxillary transverse deficiency (MTD) and those with normal transverse maxillary width.

**Material and Methods:**

A total of 111 complete cranial scans from patients aged 18 years or older with skeletal Class II malocclusion were analyzed. This classification was confirmed by measuring the ANB angle and convexity at point A. The patients were divided into two groups based on their transversal conditions: the normal transversal maxillary width group (n=46) and the atresia group (n=65). The classification followed the University of Pennsylvania (U-Penn) method using Planmeca Romexis® software. Additionally, the vertical thickness of the maxillary bone was measured at three specific points (anterior, middle, and posterior) using sagittal views. Four maxillary thickness measurements were evaluated: nasopalatine canal thickness (NCT), middle maxillary thickness (MMT), anterior maxillary thickness (AMT), and posterior maxillary thickness (PMT). Statistical analyses included Student's t-test, linear multiple regression, and Pearson correlations.

**Results:**

The maxillary atresia index showed a mean difference of 5.82 mm between the two groups (p&lt;0.001), favoring the adequate-width group. The four maxillary thickness measurements evaluated did not show significant differences between the groups (p&gt;0.05). Furthermore, a low but significant correlation was found between maxillary atresia and bone thickness in the nasopalatine canal and the posterior maxilla, specifically for NCT (R=0.202, p=0.033) and PMT (R=0.205, p=0.031). Among all the analyzed variables using multiple linear regression, only the posterior maxillary thickness (PMT) was found to influence the occurrence of atresia significantly. It was determined that for every millimeter increase in this thickness, the maxillary atresia index increased by 0.78 points (p=0.005).

**Conclusions:**

The transverse dimensions of the maxilla in individuals with skeletal Class II affect its vertical dimensions; regardless of the initial transverse condition, a narrower maxilla correlates with greater posterior palatal thickness.

## Introduction

The harmony and functionality of the stomatognathic system are fundamentals pillars for effective orthodontic treatment. However, certain conditions can affect this balance, such as maxillary transverse deficiency (MTD). Although the exact cause of posterior crossbite is not fully understood, it is considered multifactorial, involving genetic factors (skeletal or dental asymmetries), environmental factors (habits such as thumb sucking, nutrition), and functional factors (chronic mouth breathing, atypical swallowing). For these reasons, orthodontic correction becomes essential (1). The implications of MTD go beyond the development of malocclusion and aesthetic alterations and may trigger a series of complications such as inefficient mastication, improper swallowing, and even incorrect speech articulation. Additionally, it has been reported that MTD can cause changes in the mandibular condyle (2). Transverse development is crucial as it precedes vertical and sagittal growth, and alterations in this dimension can lead to issues such as impacted canines, posterior crossbites, facial asymmetry, mandibular deviations, aesthetic concerns, adverse periodontal responses, and lack of space for dental alignment (3). Several studies highlight a relationship between transverse deficiency and respiratory problems (4 , 5). A narrow maxilla, often resulting from a collapse of the respiratory system or oral breathing habits, may lead to a low tongue posture resting on the floor of the mouth. This posture influences mandibular development, resulting in a retruded mandible and a high mandibular angle, which significantly increases the risk of developing obstructive sleep apnea (OSA). MTD is usually more pronounced in the posterior region of the maxilla. In individuals with skeletal Class II malocclusion, it is typically found in the midline region of the mouth. It is more clinically significant than in other skeletal relationships, primarily due to functional and aesthetic considerations. Longitudinal studies indicate that MTD often persists into adulthood, frequently necessitating orthosurgical management (6 - 8). There are several methods for diagnosing MTD, such as clinical visualization in which posterior crown torque is observed (buccal inclination of the upper teeth and lingual inclination of the lower teeth); a "V" or "U"-shaped arch form; and a narrow palatal vault. Although useful, these are limited by dental compensation (3). Initially, diagnostic measurements were made on plaster models using analyses such as those by Pont, Korkhaus, Howe, McNamara, Mayoral, and Andrews. The posteroanterior (PA) cephalogram introduced by Ricketts in 1981 continues to be used and represents an advance in the assessment of maxillary transverse dimensions (4). In the late 1990s, the introduction of Cone Beam Computed Tomography (CBCT) in dentistry provided a significant advantage by allowing practitioners to obtain a complete three-dimensional view of maxillofacial structures. This innovation became a valuable tool for diagnosing maxillary transverse deficiency (MTD). In 2010, the University of Pennsylvania (UPenn) developed an analysis to help determine the presence of maxillary transverse deficiency. This analysis measures the widths of the maxilla and mandible, with the results indicating the extent of any transverse deficiency the patient may have. (4). Ultimately, the Yonsei Analysis (2017) (9), which also employs CBCT, determines transverse discrepancies without accounting for molar axial inclination, instead concentrating on the center of resistance of the molars, which remains unchanged. Among the treatments for MTD, rapid palatal expansion (RPE) is widely used due to its effectiveness in correcting posterior crossbites. However, it is associated with dentoalveolar effects such as buccal tipping of the maxillary molars, root resorption, bone dehiscence, and a tendency for relapse. To reduce these adverse effects, mini-implant assisted rapid palatal expansion (MARPE) has been proposed as an alternative. MARPE is used in patients with a moderate degree of skeletal maturation of the midpalatal suture, where resistance to skeletal expansion with conventional devices is considerably higher (10). It is essential to consider the patient's age when selecting treatment, as well as to ensure proper design and planning of the expansion device (11). Its use has been reported in cases requiring an increase in arch length due to anchorage loss from extractions, with a transverse correction greater than 7 mm and unilateral expansion. When the ossification of the midpalatal suture is complete, surgically assisted rapid palatal expansion (SARPE) has been described (12 , 13). MTD has been extensively studied, leading to an in-depth analysis of the horizontal dimensions of the maxilla. However, the vertical dimensions of the maxillary bone have been less explored. It has been reported that in adults with skeletal Class III, the midpalatal sutures are thinner and the width of the maxillary base is narrower compared to patients with skeletal Class I. One study reported a correlation between vertical growth patterns and the transverse dimensions of the maxilla (14). Another study mentions that individuals with a high-angle facial growth pattern are characterized by a thinner palate and a narrow, slender alveolar maxilla compared to those with a low-angle pattern (15). Recently, the study of vertical maxillary thickness (VMT) has gained importance due to the rise of therapies aimed at opening the midpalatal suture (MPS), especially in cases where ossification has begun or is already complete. The need to perform maxillary expansion is critical in cases of MTD. However, the literature is scarce regarding the potential influence of transverse deficiency on vertical maxillary thickness (VMT) in patients with skeletal Class II. Based on the above, the purpose of this study was to compare, using CBCT, the maxillary bone thickness at the level of the midpalatal suture (MPS) between skeletal Class II individuals with MTD and individuals with normal maxillary transverse width. The null hypothesis of this study is that there are no differences between the groups, and that the transverse dimensions of the maxilla do not influence its vertical dimensions.

## Material and Methods

**Figure 1:**
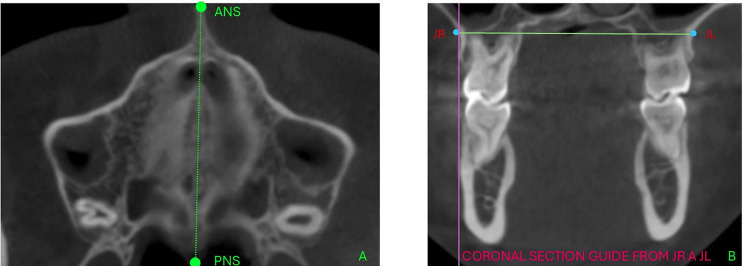
(A). Anterior Nasal Spine (ANS) and Posterior (PNS), axial view used to verify tomographic orientation. (B). Coronal section, locating the jugal points which coincide with the intersection of the lowest and most concave point of the zygomatic bone and the maxillary tuberosity on the right and left sides, points JR and JL.

This study was approved by the Research Ethics Committee of the School of Dentistry at Universidad Hemisferios, Quito, Ecuador according to Record CEUHE25-52. This descriptive, and cross-sectional study evaluated full cranial CBCT scans taken between 2022 and 2025. All scans were acquired using a Planmeca ProMax 3D Mid CBCT unit at 120 kV, 6.3 mA, with an exposure time of 18 seconds, a field of view (FOV) of 20x17 cm, and a voxel size of 0.40 mm. These scans were obtained from the tomographic database of Universidad de Los Hemisferios. A sample of 111 full cranial CBCT scans was selected. The CBCTs were divided into two groups based on their transversal conditions: the normal transversal maxillary width group (n=46) and the atresia group (n=65). The classification followed the University of Pennsylvania (U-Penn) method using Planmeca Romexis® software. The sample size for the study was determined using a formula specifically designed to compare two means. The following parameters were established: a confidence level of 95%, a statistical power of 90%, a mean difference in maxillary thickness of 1 mm, and a variance of 1 mm for the group without maxillary atresia. Based on these calculations, the minimum sample size required was 34 CT scans for each group. Inclusion criteria included scans from male and female patients aged over 18 years, with skeletal Class II, no previous orthodontic or orthopedic treatment, and presence of permanent first molars. Exclusion criteria included scans with defective images that prevented proper evaluation, or with tomographic signs of prior orthognathic or facial surgery; ongoing MARPE or SARPE treatments; or scans from individuals with syndromic conditions. To determine the skeletal class, cephalometric analysis was performed using the "NemoStudio-NemoTec" software, and classification was based on the cephalometric measurements of Ricketts' convexity at point A and Steiner's ANB angle. Based on these results, patients with other skeletal classes were excluded. The DICOM files of the CBCT scans were imported into the Planmeca Romexis® software. Reconstructions were evaluated considering cephalic orientation in all three spatial planes. The selection of slices for the evaluation of MTD and maxillary vertical bone thickness followed the protocol below: initially, in the sagittal view, the software cursor was positioned on the patient's midsagittal plane, aligned with the anterior nasal spine (ANS) and posterior nasal spine (PNS). Next, in the coronal slice of the upper maxilla, the jugal points were located, corresponding to the intersection of the most inferior and concave point of the zygomatic bone and the maxillary tuberosity on both the right and left sides (Fig. 1).

1

**Figure 2:**
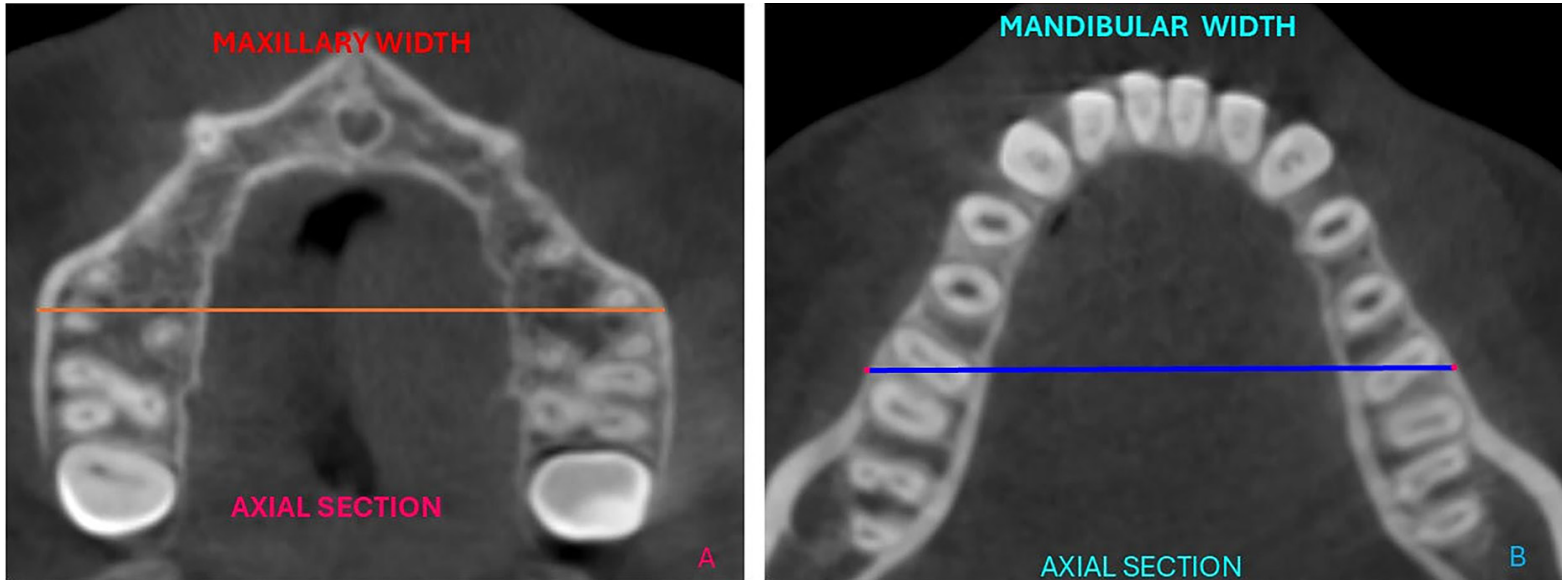
(A). Axial section, measurement of maxillary width. (B). Axial section, measurement of mandibular width.

For the measurement of mandibular width in the coronal plane, the level of the furcations of the lower first molars was used, which corresponds to the point where the roots diverge. This position was maintained as a cutting guide, and in the axial slice, using the software's measurement tool, the mandibular and maxillary widths were measured at the level of the furcation and intersection of the cortical bone corresponding to the outer cortical plates of the first molars, from right to left, with values recorded in millimeters (Fig. 2).

2

**Figure 3:**
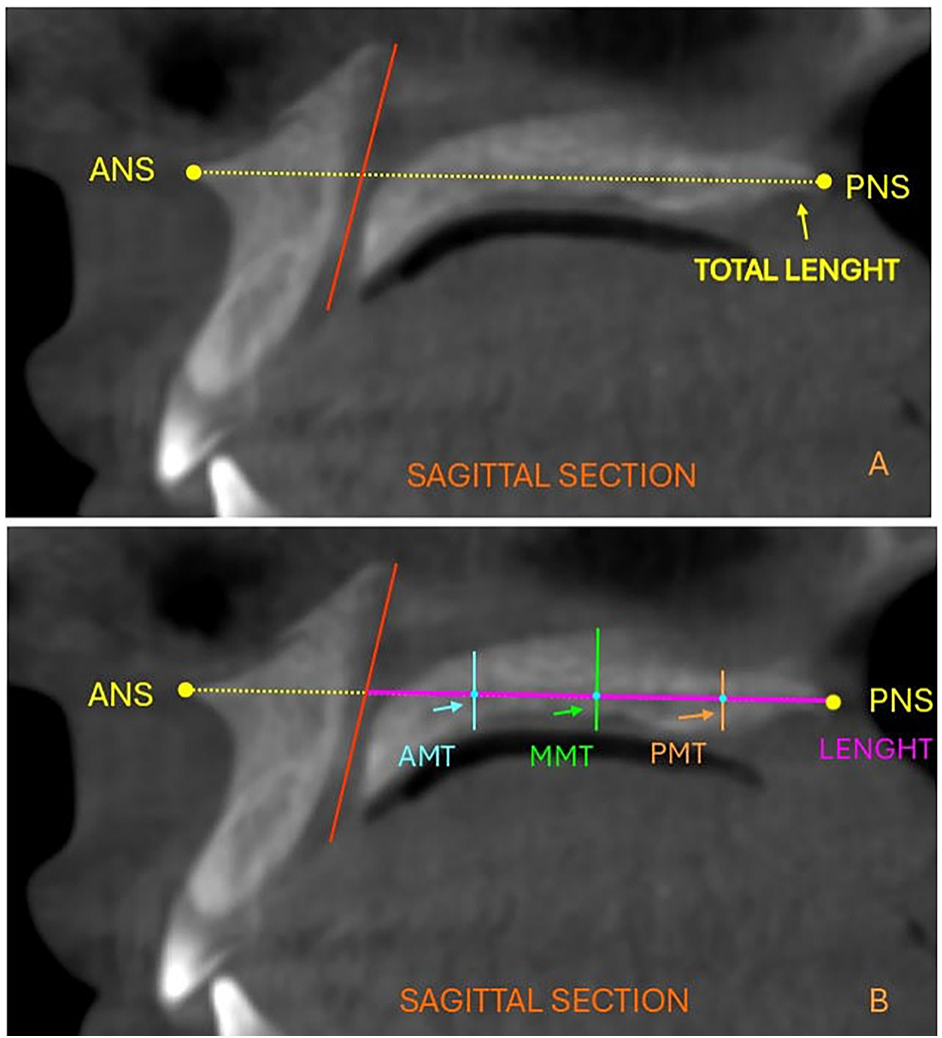
(A). Length of the maxillary plane from ANS to PNS and plane of the nasopalatine canal. (B). Anterior maxillary thickness (AMT), middle maxillary thickness (MMT), and posterior maxillary thickness (PMT) perpendicular to the maxillary plane.

According to the U Penn method, 5 mm was added to the mandibular width value as a constant, which represents the ideal average difference between the maxillary and mandibular widths required for correct occlusion. Then, the resulting value was subtracted from the maxillary width value, determining the difference between them. Maxillary and mandibular widths, along with skeletal classification. The evaluator was trained and calibrated by an experienced orthodontist and radiologist specialized in CBCT evaluation and measurement. All measurements were performed twice by the same evaluator, with a 30-day interval between sessions. Intraobserver agreement for the quantitative variables was assessed using the intraclass correlation coefficient (ICC), calculated with STATA version 16, yielding a value of 0.90, interpreted according to the Landis and Koch scale (16). In the sagittal view, a line was drawn from the anterior nasal spine (ANS) to the posterior nasal spine (PNS) to define the maxillary plane, and its length was measured. At the level of the nasopalatine canal, a line connecting the center of the canal on the palatal side to its center at the nasal floor was drawn. This measurement was referred to as the vertical length of the nasopalatine canal (NCT) (Fig. 3).

3

From this line, the distance to the PNS was measured, and the midpoint along the maxillary plane was identified. At this midpoint, the central maxillary thickness (MMT) was measured perpendicularly to the maxillary plane. From the intersection of MMT with the maxillary plane, two additional points were established: the anterior maxillary thickness (AMT) and the posterior maxillary thickness (PMT). The AMT was located at the midpoint between NCT and MMT, while the PMT was positioned at the midpoint between MMT and PNS. Maxillary thickness measurements were then taken at these points, also perpendicular to the maxillary plane (Fig. 3). - Statistical Analysis All data were recorded in an Excel spreadsheet, and statistical analyses were performed using SPSS for Windows (version 26; IBM, Armonk, NY). The normality of the data was verified with the Shapiro-Wilk test. Intergroup comparisons were conducted using the Student's t-test for independent samples. To assess the relationships between maxillary width, maxillary length, and maxillary thickness, Pearson's correlation test was applied. Finally, multiple linear regression was used to evaluate the influence of predictive variables on the maxillary width index. A statistical significance level of p&lt;0.05 was set for all tests.

## Results

Table 1 describes the initial characteristics of the maxillae in each group, classified according to their transverse maxillary condition.

Table 1

Significant differences were found in maxillary width (p &lt; 0.001), with a mean difference of 3.78 mm in favor of the adequate-width group, and in the maxillary atresia index (p &lt; 0.001), with a mean difference of 5.82 mm also in favor of the adequate-width group. Also, the four maxillary thickness measurements -nasopalatine canal thickness, middle maxillary thickness, anterior maxillary thickness, and posterior maxillary thickness- evaluated did not show significant differences between the groups (p&lt;0.05). Table 2 shows the correlation between the maxillary atresia index and both the palatal plane length and the evaluated maxillary thicknesses, revealing low but statistically significant correlations in two specific thicknesses: NCT (R = 0.202, p = 0.033) and PMT (R = 0.205, p = 0.031).

Table 2

Table 3 summarizes the multiple linear regression analysis on the influence of predictive variables, including palatal plane length, maxillary thicknesses, and others on the maxillary atresia index.

Table 3

Only posterior maxillary thickness (PMT) had a significant influence: for every 1 mm increase in PMT, the maxillary atresia index increased by 0.78 points (p = 0.005).

## Discussion

The current study used CBCT to compare the thickness of the maxillary bone at the midpalatal suture (MPS) between two groups of individuals with skeletal Class II malocclusion. One group exhibited MTD, while the other group had a normal maxillary transverse width. The analysis showed a mean difference of 3.78 mm (p &lt; 0.001) in maxillary width between the groups. Based on the maxillary atresia index, there was a mean difference of 5.82 mm, again favoring the group with adequate width (p &lt; 0.001). The results indicated no significant differences in the four maxillary thickness measurements-(NCT, MMT, AMT, and PMT)-between the two groups (p &lt; 0.05). However, when the two groups were combined, a positive correlation was observed between the vertical length of the nasopalatine canal, posterior maxillary thickness, and the maxillary atresia index. Specifically, as the thickness of the palatal bone increases at the level of the nasopalatine canal and in the posterior region, the atresia index also rises. This indicates that the narrower the maxilla is transversely, the greater the thickness of the posterior palatal bone. This finding is clinically important as it provides an additional criterion for more precise and appropriate selection of mini-implants in cases of MTD where MARPE or SARPE is planned. A greater thickness at the MPS implies more resistance to expansion, which in turn would require mini-implants with a larger diameter in the posterior maxillary region to ensure sufficient torque and increased contact surface.The results of this study also suggest that the mini-implants used in the anterior palatal region could have a smaller diameter than those used posteriorly. These findings are consistent with those reported by Li et al. (17) who evaluated vertical maxillary thicknesses across different skeletal classes and found greater thickness in skeletal Class II individuals. However, their study did not include patients with maxillary atresia, which reinforces the relevance of our findings. Several studies have evaluated vertical maxillary thickness. Ning et al. (15) reported that individuals with high-angle facial patterns have thinner palatal bone compared to those with low-angle patterns, regardless of sex; however, their study focused on evaluating cranio-maxillofacial morphological differences between males and females. Chen et al. (18) assessed palatal bone thickness in areas designated for skeletal maxillary expander placement in adult Class III patients and reported that, in general, it was thinner compared to patients with skeletal Class I. Morphological differences in maxillary thickness between skeletal classes are a relevant finding, particularly in Class II patients with MTD (20). Wang et al. (14 , 19) analyzed 72 palatal areas in 51 adolescents and 56 adults, aiming to measure bone thickness in each participant without considering skeletal class or transverse maxillary dimensions. They found that, in the sagittal plane, palatal bone thickness progressively decreased from a reference line posteriorly from 0 to 9 mm in adults and from 0 to 12 mm in adolescents, findings that do not align with those of the present study. The skeletal classification of each individual requiring orthodontic treatment is an important diagnostic factor in treatment planning, but it becomes especially relevant in patients with MTD. Additionally, the positive correlation found with the vertical length of the nasopalatine canal in these cases supports the need for a detailed tomographic study of this area when considering maxillary expansion, whether in a growing patient using conventional rapid expansion mechanics such as a Hyrax appliance, or when the use of skeletal anchorage, like mini-implants, is indicated in SARPE or MARPE procedures. Another interesting finding from the present study, derived from multiple linear regression, was that posterior maxillary thickness (PMT) is a factor influencing the maxillary atresia index. Our findings indicate that for every millimeter increase in posterior maxillary thickness, the maxillary atresia index increases by 0.78 points. This suggests that the greater the severity of the transverse maxillary deficiency, the more challenging its skeletal correction will be, as the posterior maxillary bone will be thicker, requiring greater force and torque during maxillary expansion if palatal skeletal anchorage is used. The findings reported here are also relevant for the selection of mini-implants in cases requiring skeletal anchorage without maxillary expansion, where the sutural region is chosen as the placement area, such as in cases of distalization of maxillary molars and premolars (20 , 21), or in cases with strict anchorage requirements where mini-implants are placed in the midpalatal suture (22). The null hypothesis of this study is partially rejected, as no differences were found between the groups. However, the transverse maxillary dimensions do influence the vertical dimensions. One limitation of this study is the sample size. However, statistically significant correlations were identified; a larger sample could yield more robust results and allow for a more detailed subgroup analysis. Another significant limitation is the method used for diagnosing transverse maxillary deficiency. While the UPenn method is a validated and reliable protocol, it relies on a constant that may not account for the anatomical variability among patients, potentially affecting diagnostic accuracy. As recommendations, the study suggests conducting prospective and longitudinal studies to monitor patients over time, which could help establish cause-and-effect relationships. Additionally, it would be beneficial to evaluate whether palatal dimensions change in response to orthodontic treatments. Further analysis of different correlations between transverse maxillary deficiency and other variables, such as the ossification of the midpalatal suture and the patients' age, is needed to determine factors influencing the severity of maxillary atresia.

## Conclusions

The assessment of maxillary thickness showed no significant differences between groups with maxillary transverse deficiency and those with normal transverse maxillary width. In individuals with skeletal Class II, the transverse dimensions of the maxilla influence its vertical dimensions. Specifically, a narrower maxilla, regardless of its initial transverse condition, is associated with greater posterior palatal thickness.

## Figures and Tables

**Table 1:** Initial characteristics of the maxillae in each group evaluated according to their transverse maxillary condition.

Variable	Transverse MaxillaryCondition	n	Mean	SD	P-value	Mean difference	95% Confidence Interval of the Difference	
							Lower	Upper
Age	Normal	46	28.30	8.54	0.036*	-4.08	-7.89	-0.27
	Atresia	65	32.38	10.88				
SNA	Normal	46	83.67	5.03	0.442	0.65	-1.03	2.35
	Atresia	65	83.02	3.96				
SNB	Normal	46	77.28	5.24	0.449	0.66	-1.07	2.4
	Atresia	65	76.62	4.01				
ANB	Normal	46	6.29	1.71	0.702	0.13	-0.55	0.82
	Atresia	65	6.16	1.85				
Convexity	Normal	46	5.95	2.2	0.643	0.2	-0.66	1.06
	Atresia	65	5.75	2.29				
Maxillary Width	Normal	46	61.83	3.9	>0.001*	3.78	2.42	5.15
	Atresia	65	58.04	3.33				
Mandibular Width	Normal	46	53.92	2.99	0.288	-9.63	-27.51	8.26
	Atresia	65	63.55	61.06				
Maxillary Atresia Index	Normal	46	2.9	2.12	>0.001*	5.82	4.93	6.71
	Atresia	65	-2.92	2.47				
ANS-PNS	Normal	46	37.52	2.28	0.228	0.61	-0.39	1.62
	Atresia	65	36.9	2.86				
NCT	Normal	46	13.73	2.17	0.125	0.64	-0.18	1.47
	Atresia	65	13.09	2.15				
MMT	Normal	46	7.34	1.46	0.988	0	-0.6	0.59
	Atresia	65	7.34	1.62				
AMT	Normal	46	6.61	1.35	0.822	0.06	-0.5	0.63
	Atresia	65	6.55	1.56				
PMT	Normal	46	6.97	1.52	0.082	0.51	-0.06	1.08
	Atresia	65	6.47	1.48				

*Significant, Student’s t-testANS-PNS = Palatal plane lengthNCT = Nasopalatine canal thicknessMMT = Middle maxillary thicknessAMT = Anterior maxillary thicknessPMT = Posterior maxillary thickness

**Table 2:** Correlations between the maxillary width index and the palatal plane length and maxillary bone thicknesses.

Variables		ANS-PNS	NCT	MMT	AMT	PMT
Maxillary Width Index	Pearson’s R	0.183	0.202	-0.012	0.051	0.205
	p-value	0.055	0.033*	0.900	0.594	0.031*
	n	111	111	111	111	111

*SignificantANS-PNS = Palatal plane lengthNCT = Nasopalatine canal thicknessMMT = Middle maxillary thicknessAMT = Anterior maxillary thicknessPMT = Posterior maxillary thickness

**Table 3:** Multiple linear regression evaluating the influence of predictive variables on the maxillary width index.

Variable	B	P	95.0% confidence interval for B	
			Lower limit	Upper limit
ANS-PNS	0.21	0.128	-0.06	0.47
NCT	0.32	0.066	-0.02	0.66
MMT	-0.42	0.155	-1018.00	0.16
AMT	-0.02	0.940	-0.57	0.53
PMT	0.78	0.005*	0.23	1328.00
Sex	-0.79	0.316	-2347.00	0.77
Age	-1086.00	0.122	-2468.00	0.29
ANB	0.23	0.218	-0.14	0.60

*SignificantANS-PNS = Palatal plane lengthNCT = Nasopalatine canal thicknessMMT = Middle maxillary thicknessAMT = Anterior maxillary thicknessPMT = Posterior maxillary thickness

## Data Availability

The datasets analyzed in this study can be obtained from the corresponding author.
